# Numerical research of the influence mechanism of fan diameter and jet velocity on tunnel ventilation efficiency

**DOI:** 10.1038/s41598-026-53723-w

**Published:** 2026-05-21

**Authors:** Wenjiao You, Jie Kong, Shuli Li, Yixuan Zhang, Yimeng Cao

**Affiliations:** 1 Laboratory of Public Safety Risk Governance, Zhejiang Police College, Hangzhou, 310053 China; 2 Department of Public Security, Zhejiang Police College, Hangzhou, 310053 China; 3 College of Energy Environment and Safety Engineering, China Jiliang University, Hangzhou, 310018 China

**Keywords:** Tunnel ventilation, Jet fan, Jet characteristics, Ventilation efficiency, Ventilation energy saving, Energy science and technology, Engineering

## Abstract

Full-jet longitudinal ventilation is widely used in urban tunnels for its structural simplicity and high cost-effectiveness, yet its overall efficiency is constrained by high energy consumption. This study systematically investigates the influence of jet fan diameter and outlet velocity on ventilation performance. A three-dimensional numerical model of a typical urban tunnel was developed using ANSYS Fluent. Parametric simulations were conducted by varying jet velocity from 20 m/s to 38 m/s and fan diameter from 500 mm to 1600 mm. Jet flow evolution and the underlying mechanisms governing ventilation efficiency, including pressure development, air entrainment, and cross-sectional flow uniformity were rigorously analyzed. Results indicate that for a 500 mm diameter fan, increasing jet velocity from 20 m/s to 38 m/s elevates the ventilation pressure coefficient from 16% to 66%. At a fixed velocity of 38 m/s, enlarging the diameter from 500 mm to 1600 mm further improves the pressure enhancement coefficient from 66% to 73%, with fan pressure gain exhibiting a quadratic relationship with diameter. The analysis demonstrates that higher jet velocities enhance both momentum thrust and ambient air entrainment, thereby strengthening the longitudinal airflow driving force. Concurrently, larger fan diameters widen the jet diffusion angle, producing a more uniform cross-sectional airflow with reduced stratification. These findings clarify the distinct yet interdependent roles of jet velocity and fan diameter in jet behavior and ventilation efficacy, offering quantitative guidance for fan selection and energy-efficient longitudinal ventilation design.

## Introduction

In recent years, a large number of tunnels have been built to relieve the traffic pressure in the city^1,2^. Vehicles passing through the tunnel will retain a large amount of harmful gases such as CO, NOx, HC, and particulate matter (PM), posing a threat to personnel health and driving safety^3–5^. In addition, the substantial electricity consumption of ventilation systems has become a major operational cost. Therefore, enhancing ventilation efficiency and reducing energy consumption through system optimization and fan parameter adjustments are key research objectives in modern tunnel engineering^6–8^.

The ventilation system constitutes an integral component of the tunnel safety system. Under routine operating conditions, it serves to effectively ameliorate the air quality and driving environment within the tunnel^9,10^. In event of fire, the system is designed to control and extract smoke, extending the available safe evacuation time for occupants^11^. The design of the highway tunnel ventilation system must strike a balance between cost-effectiveness and other factors such as tunnel length, traffic conditions, climate, topography and geology. Commonly used tunnel ventilation methods encompass natural ventilation^12^, transverse ventilation^13^, semi-transverse ventilation^14^, full-jet longitudinal ventilation and combined ventilation^15^. With advances in construction technology, ventilation has emerged as a critical constraint on the development of tunnel engineering. Among various ventilation strategies, full-jet longitudinal ventilation is widely adopted in urban tunnels owing to its cost-effectiveness and operational simplicity^10,16^.

Numerous factors affect the jet fan ventilation efficiency, including fan arrangement, tunnel geometry, air temperature, air humidity, jet flow speed, and fan diameter. Since 2000, research efforts have primarily centered on quantifying the impact of fan arrangement on tunnel ventilation efficiency. Widely adopted investigative approaches include Computational Fluid Dynamics (CFD) simulations, scaled modeling, and field measurements.

The jet flow generated by highway tunnel fans is a complex turbulent phenomenon, where the continuity equation and Navier-Stokes equations serve as the core control equations of computational fluid dynamics. Theoretically, Direct Numerical Simulation (DNS) can directly resolve turbulent flows across all scales without the need for turbulence modeling. However, due to its extremely high computational cost, Reynolds-averaged Navier-Stokes (RANS) equations were developed as a practical alternative for engineering applications. The time-average equations of continuity and momentum equations are given as Eqs. (1) and (2).

1 $$\frac{{\partial {{\bar {u}}_i}}}{{\partial {x_i}}}=0$$

2 $$\rho \frac{{\partial {{\bar {u}}_i}}}{{\partial t}}+\rho {\bar {u}_j}\frac{{\partial {{\bar {u}}_i}}}{{\partial {x_j}}}= - \frac{{\partial \bar {p}}}{{\partial {x_i}}}+\frac{\partial }{{\partial {x_j}}}\left[ {\mu \left( {\frac{{\partial {{\bar {u}}_i}}}{{\partial {x_j}}}+\frac{{\partial {{\bar {u}}_j}}}{{\partial {x_i}}}} \right) - \rho \overline {{{{u^{\prime}}_i}{{u^{\prime}}_j}}} } \right]+\rho {\bar {g}_i}$$

Both the *k-ε* and k-ω turbulence models belong to the two-equation eddy viscosity model family under the RANS framework. Specifically, the *k-ε* model is characterized by strong robustness, stable computation, high efficiency, and high simulation accuracy for fully developed turbulence^17^. These advantages make it particularly suitable for simulating fully developed pipe flows and free jets without wall interference. In contrast, the *k-ω* model exhibits high simulation accuracy for wall boundary layers, flow separation, and inverse pressure gradient flows, making it more applicable to scenarios involving jet-wall interactions (e.g., tunnel jet fan ventilation). The standard *k-ε* model has been widely adopted by researchers in the field of tunnel ventilation^10,18–20^. The transport equations of k and ε in the standard *k-ε* model are shown as Eqs. (3) and (4).

3 $$\frac{\partial }{{\partial t}}\left( {\rho k} \right)+\frac{\partial }{{\partial {x_i}}}\left( {\rho k{u_i}} \right)=\frac{\partial }{{\partial {x_j}}}\left[ {\left( {\mu +\frac{{{\mu _t}}}{{{\sigma _k}}}} \right)\frac{{\partial k}}{{\partial {x_j}}}} \right]+{G_k} - \rho \varepsilon$$

4 $$\frac{\partial }{{\partial t}}\left( {\rho \varepsilon } \right)+\frac{\partial }{{\partial {x_i}}}\left( {\rho \varepsilon {u_i}} \right)=\frac{\partial }{{\partial {x_j}}}\left[ {\left( {\mu +\frac{{{u_t}}}{{{\sigma _\varepsilon }}}} \right)\frac{{\partial \varepsilon }}{{\partial {x_j}}}} \right]+{C_{{\varepsilon _1}}}\frac{\varepsilon }{k}{G_k} - {C_{{\varepsilon _2}}}\frac{{{\varepsilon ^2}}}{k}\rho$$

Weng et al.^21^ employed Fire Dynamics Simulator (FDS) software to develop a ventilation model of the Zhong Liangshan Tunnel and investigated the effects of varying jet fan spacings on the temperature and velocity fields. By comparing and analyzing the velocity and temperature distributions for fan spacings of 100 m, 150 m, and 200 m, the optimal configuration was identified as a fan spacing of 200 m. The numerical simulation results show excellent agreement with experimental data obtained from a reduced-scale physical model, validating their use for assessing jet fan ventilation performance in tunnels. Key factors considered in the investigation are installation height, spacing, distance from tunnel exit, optimal pitch angle^22,23^, and deflection angle^18^. Fan et al.^24^ observed that jet fan placement exhibits considerable flexibility in short tunnels. In contrast, for long tunnels, particularly underwater, mountain, or super-long urban tunnels, a series configuration of jet fan units is typically adopted. Longitudinal multi-group and cross-sectional multi-jet fan arrangements significantly affect tunnel ventilation performance and efficiency. Wang et al.^10^ used the CFD software Fluent to study jet fan ventilation in curved tunnels. The energy loss caused by the interaction between high speed air jet and curved wall leads to a decrease in ventilation efficiency.

To enhance the tunnel environment and investigate the airflow development characteristics induced by jet fans, Zhang et al.^25^ conducted both 1:25 scale model tests and CFD simulations. These studies analyzed key aerodynamic parameters, including wind speed, wind pressure, turbulence intensity, and ventilation effectiveness equipped with varying numbers of parallel jet fans. Results indicate that, at a jet fan outlet velocity of 20 m/s, the homogenized airflow speed in the tunnel reaches 1.3 m/s for a single fan, 1.9 m/s for two parallel fans, and 2.5 m/s for three parallel fans. Furthermore, the authors derived an attenuation equation of the longitudinal wind speed in the tunnel:

5 $${{{u_m}} \mathord{\left/ {\vphantom {{{u_m}} {{u_0}}}} \right. \kern-0pt} {{u_0}}}=1.38{\left( {{x \mathord{\left/ {\vphantom {x {{b_0}}}} \right. \kern-0pt} {{b_0}}}} \right)^{ - 0.5}}$$

Comprehensively considering parameters influencing tunnel ventilation performance, including the number of parallel fans, outlet jetflow velocity, and fan diameter, the installation of two parallel jet fans is demonstrated to improve driver comfort while concurrently reducing ventilation energy consumption. For a three-lane tunnel with a width of 12.50 m, it is recommended to arrange two parallel fans with the diameter of 1458 mm and the outlet velocity of 25 m/s in each section.

To optimize the performance parameters of jet fans, it is necessary to analyze the aerodynamic characteristics of the jetflow^19,26^. Jet fans achieve momentum transfer by injecting high speed airflow, thereby inducing longitudinal airflow within tunnels and increasing the air velocity to achieve effective ventilation. In this process, the influence of fan diameter and jet velocity on ventilation efficiency will be studied first.

In general, existing research on improving tunnel ventilation efficiency has predominantly relied on fixed fan models, focusing on the adjustment of parameters such as fan spacing, tunnel geometry, and inclination angle. However, insufficient attention has been paid to the inherent jet capacity of individual fans and the fundamental factors governing flow-induced effects, which are critical prerequisites for optimizing fan arrangement. Through the investigation of key parameters such as outlet velocity and fan diameter, the understanding of jet flow characteristics and mechanisms of performance optimization can be enhanced, providing support for fan selection and layout optimization, thereby contributing to the energy-efficient design of tunnel ventilation systems. The findings are expected to help achieve the goals of energy conservation and carbon reduction, while also improving tunnel operational safety and environmental quality.

## Establishment and verification of tunnel ventilation model

### Establishment of tunnel ventilation model

**Fig. 1:**
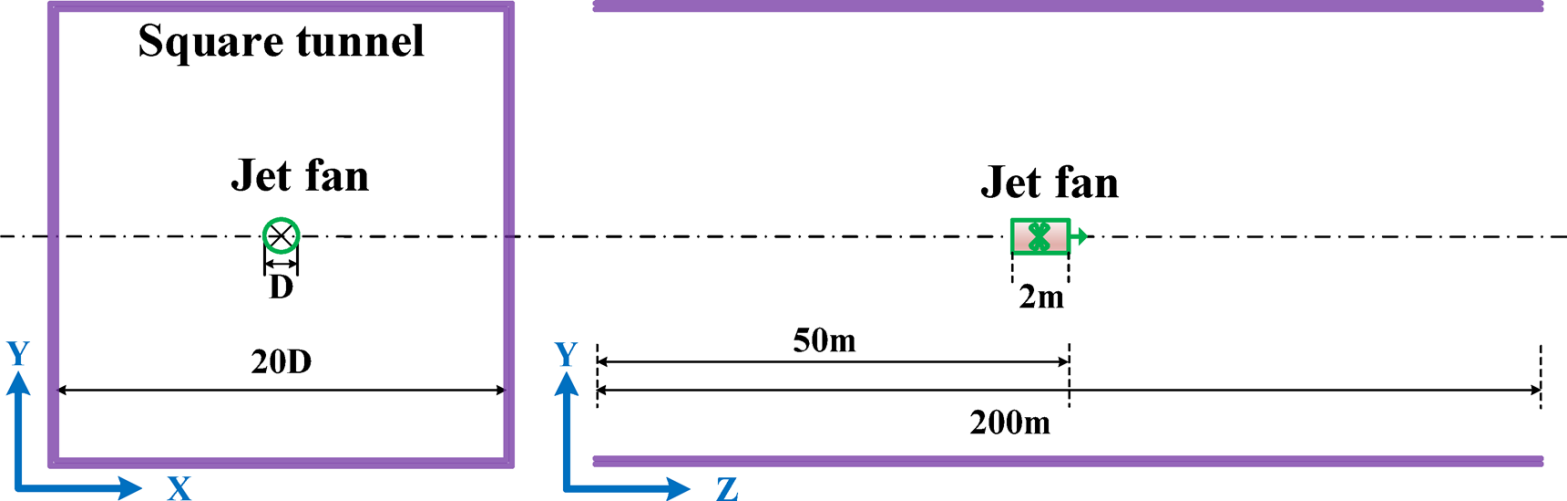
Schematic of the jet ventilation tunnel model.

In this study, a numerical model for jet flow development is established using ANSYS 2020 R2 Fluent software, and the standard *k-ε* two-equation model was selected for the turbulence model^27^. A schematic of the model is presented in Fig. 1, and the corresponding parameters are detailed in Table 1. The investigation considers 10 different fan diameters. For the 500 mm diameter series, outlet velocities range from 20 m/s to 38 m/s. When a fan-generated jet impinges on a wall or obstacle during its development, it undergoes abrupt deflection, leading to vortex formation that dissipates pressure energy and reduces ventilation efficiency. To minimize the influence of tunnel geometry, equipment obstacles, and wall friction, this study specifically focuses on the effects of fan performance parameters, the influence of fan size and jet outlet velocity on ventilation efficiency. The model employs a square cross-section tunnel with a width set to 20 times the fan diameter.

**Table 1:** Operating conditions.

Condition number	D(Fan diameter/mm)	L(Tunnel width/m)	H(Tunnel height/m)	Fan exit position(m)	Fan outlet velocity (m/s)
Da-Dj	500	10.0	10.0	50	20,22,24,26,28,30,32,34,36,38
Da2	630	12.6	12.6		38
Da3	710	14.2	14.2		38
Da4	800	16.0	16.0		38
Da5	900	18.0	18.0		38
Da6	1000	20.0	20.0		38
Da7	1120	22.4	22.4		38
Da8	1250	25.0	25.0		38
Da9	1400	28.0	28.0		38
Da10	1600	32.0	32.0		38

In the tunnel ventilation system employing full-jet fans, air is modeled as an incompressible, homogeneous viscous fluid. During the flow process, frictional dissipation occurs both between fluid layers and at the tunnel walls. The jets produced by the fans exhibit both laminar and turbulent states, resulting in highly complex and unsteady flow fields. This study employs fundamental fluid dynamics equations for ventilation analysis.

### Grid setting and model verification

In numerical simulations, the grid density and boundary conditions significantly influence the results. Following grid independence verification, the tunnel grid size was set to 0.4 m, with refinement to 0.1 m in the fan and surrounding regions, as detailed in Table 2.

The purpose of this paper is to investigate the pressure rise coefficient of jet fans in tunnels with the same design wind speed. Simplifications and assumptions were made for the research model and scenarios in this study, with the relevant explanations elaborated as follows: firstly, the length of the tunnel model adopted for the ventilation efficiency analysis was set to 200 m; secondly, the ventilation in the tunnel will eventually stabilize at the design wind speed; thirdly, jet fans actually operate with the longitudinal ventilation environmentl; fourth, the tunnel entrance was set as a velocity inlet to provide a design wind speed of 4 m/s. Besides, the boundary conditions and the design wind speed refer to the previous similar studies on tunnel ventilation. The outlet was defined as a pressure outlet.

The k-ε model is characterized by strong robustness, stable computation, high efficiency, and high simulation accuracy for fully developed turbulence. Chen et al.^27^ conducted comparative validation of the standard *k-ε* model, RNG *k-ε* model, and realizable *k-ε* model using a 1:20 scaled tunnel ventilation model. The results indicated that the simulation results of the standard *k-ε* model showed the best consistency with the experimental data. Therefore, the standard *k-ε* model was ultimately selected as the CFD turbulence analysis model in this paper.

**Table 2:** Grid parameter setting.

Condition number	D(Fan diameter /mm)	L(Tunnel width /m)	H(Tunnel height /m)	Outlet velocity (m/s)	Number of grid cells
Da-Dj	500	10.0	10.0	20; 22; 24; 26; 28; 30; 32; 34; 36; 38	432,615
Da2	630	12.6	12.6	38	666,720
Da3	710	14.2	14.2		864,735
Da4	800	16.0	16.0		1,088,460
Da5	900	18.0	18.0		1,337,895
Da6	1000	20.0	20.0		1,730,460
Da7	1120	22.4	22.4		1,730,460
Da8	1250	25.0	25.0		2,520,620
Da9	1400	28.0	28.0		3,129,340
Da10	1600	32.0	32.0		4,353,840

**Fig. 2:**
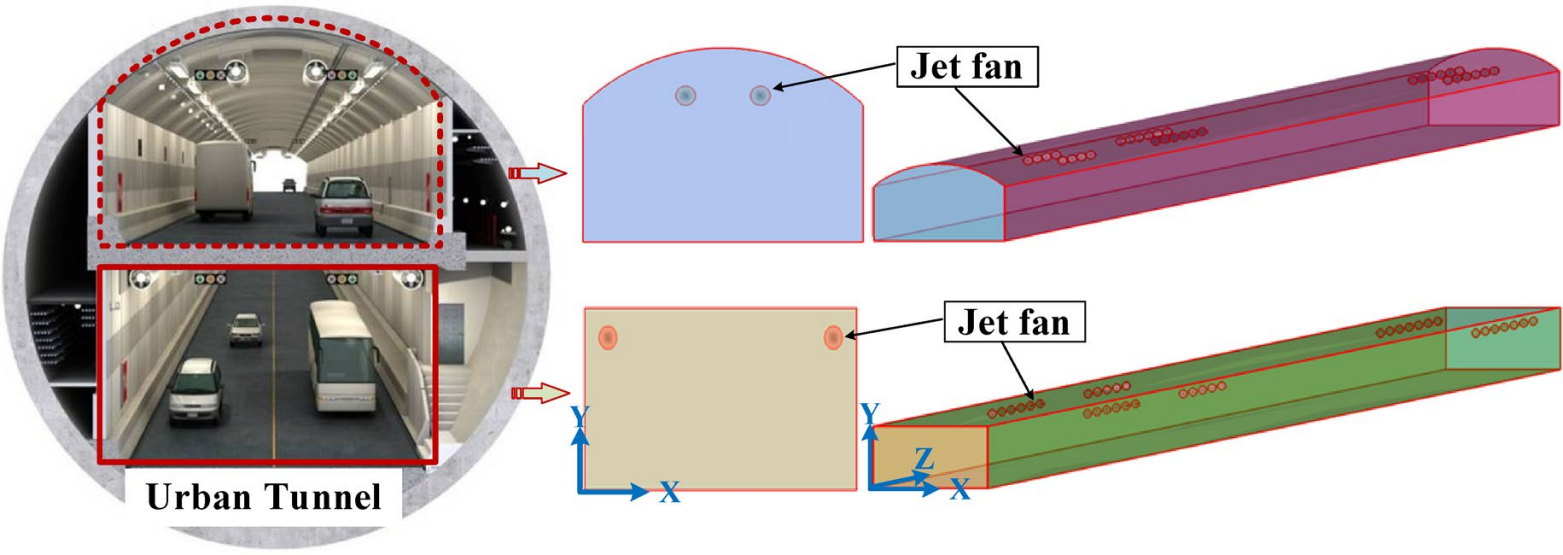
Schematic of the jet ventilation tunnel model.

**Fig. 3:**
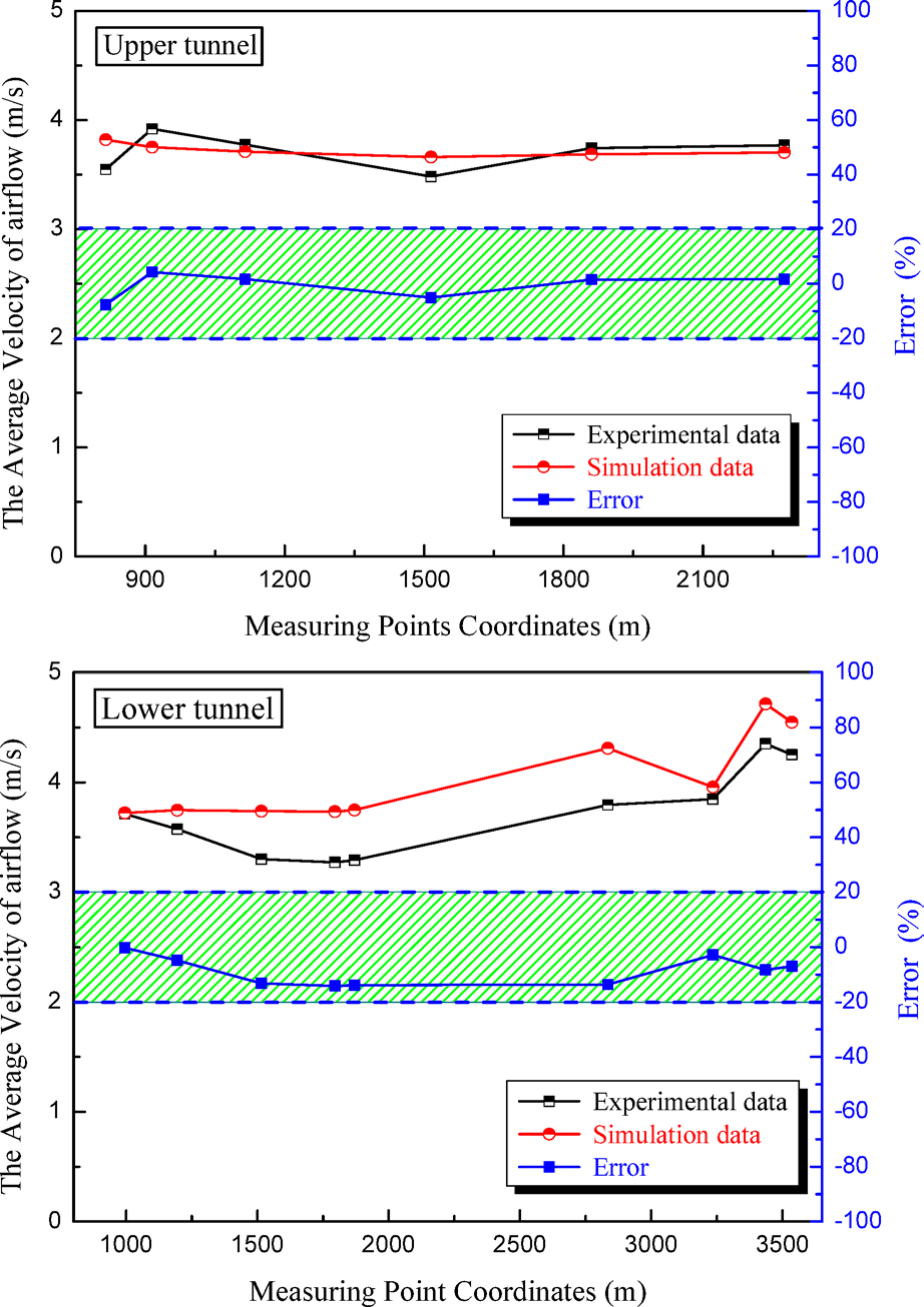
Comparison of tunnel ventilation test and simulation results.

To validate the feasibility of tunnel ventilation via numerical simulation, a 1:1 full-scale model was developed based on a specific river-crossing tunnel. Numerical simulations were compared with field ventilation tests, as illustrated in Figs. 2 and 3. The airflow velocity data for the upper and lower layers of the tunnel were obtained using a multi-channel hot wire anemometer measurement system^26^. Parameters of the measurement system are shown in Table 3. The experimental measurements for the upper layer showed good agreement with the numerical results, with the deviation maintained within 10%. Both the field tests and numerical simulations exhibited consistent performance.

**Table 3:** Main parameters of sensors.

Items	Parameters
Supply voltage	DC 24 V ± 20% max. 50 mA
Permitted ambient temperature	−5∼60 °C
Sensing range	0.2∼10 m/s
Accuracy (5∼45 °C and 1013 hPa)	± (0.3 m/s + 5% from the MW)
Settling time (after applying the supply voltage)	≤ 20 s
Settling time (at a temperature jump of 10 K)	≤ 3 min

Some discrepancies were observed between experimental data and numerical simulations in the lower tunnel level. Field investigations revealed that this section has a smaller cross-sectional area, leading to higher ventilation resistance, as well as potential air leakage into smoke ducts or emergency exits. Nevertheless, the experimental results for the lower tunnel remain within a 20% error margin compared to the numerical simulation, confirming the feasibility of this approach for studying ventilation and airflow distribution in tunnels.

## Results and analysis

### The influence of fan outlet velocity on tunnel ventilation

#### Jet velocity Distribution

**Fig. 4:**
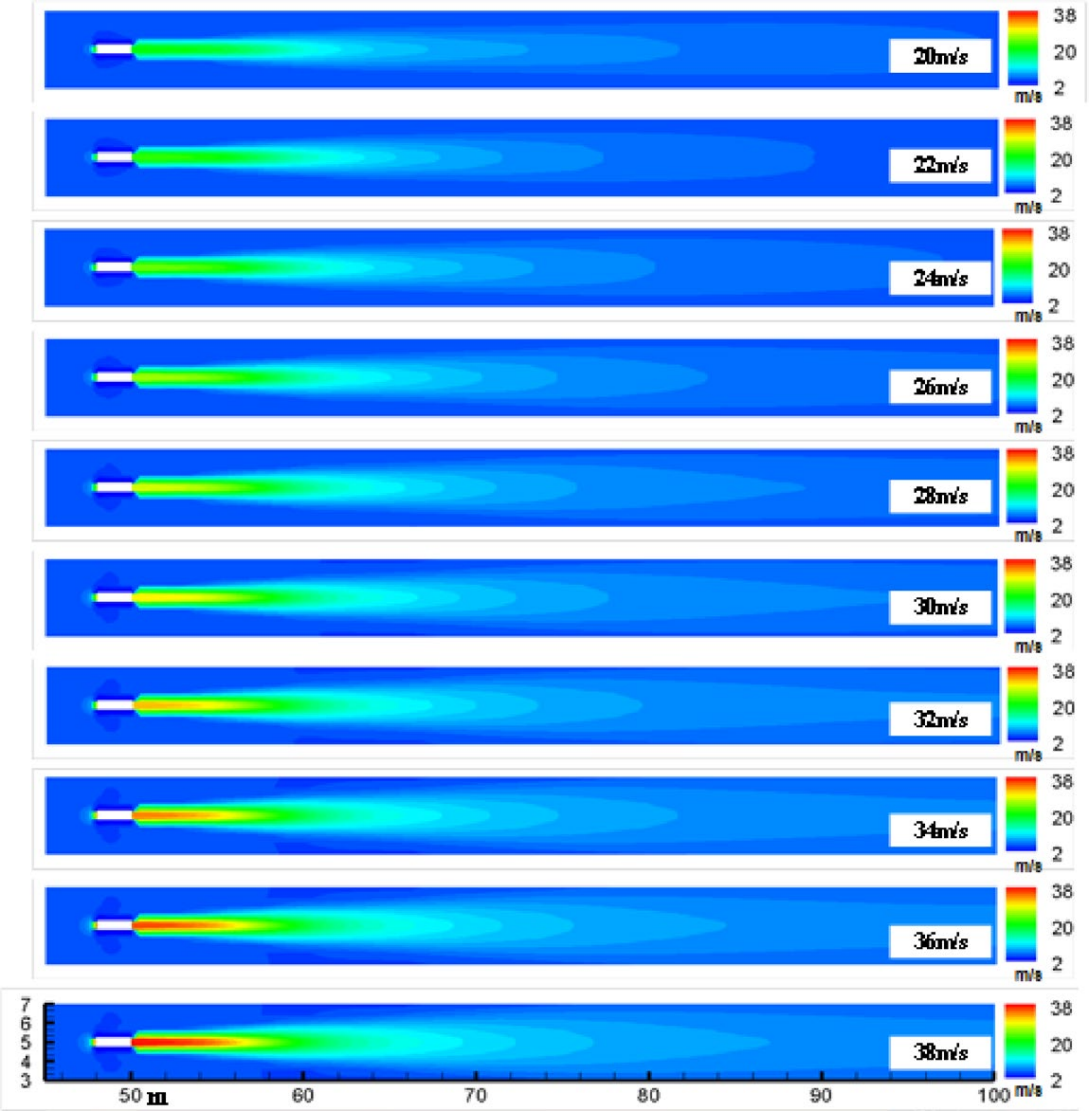
Velocity contours in the tunnel under varying fan outlet velocities.

Based on the simulation results, velocity contours under varying fan outlet velocities were obtained for the vertical plane (X = 3–7 m) and longitudinal section (Z = 45–100 m) of the tunnel, as shown in Fig. 4. As the outlet velocity increases, the jet extends further into the tunnel, accompanied by an expansion in both its axial influence range and lateral spread. This enables the ventilation airflow to maintain effective thrust over extended distances, thereby demonstrating improved ventilation capacity and operational efficacy.

Furthermore, velocity contours show a region of reduced airflow speed near the fan compared to the ambient, with the velocity deficit widening at higher outlet velocity. This is attributed to the pressure drop induced by the jet flow between the intake and exhaust. The conversion of pressure potential energy to kinetic energy is thus constrained, resulting in suppressed local air velocities.

**Fig. 5:**
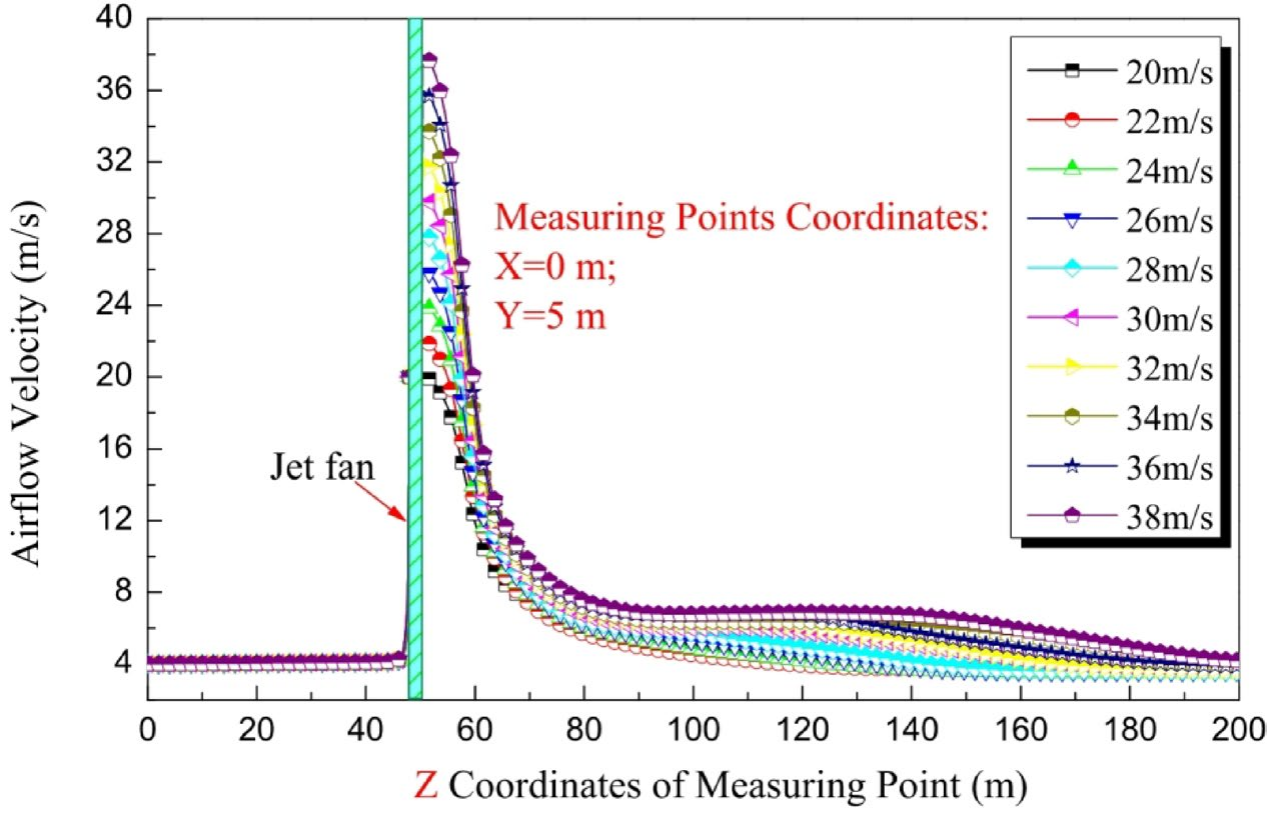
Longitudinal airflow velocity variation at the fan centerline height.

Figure 5 shows the longitudinal velocity profiles at the centerline of the 500 mm fan for outlet velocities ranging from 20 to 38 m/s. In the region from 0 to 48 m downstream, the velocities across all profiles are similar, suggesting that the jet’s effect is minimal within this range. Higher outlet velocities lead to increased peak values in the profiles, indicating that greater discharge speeds generate stronger airflow at identical downstream locations, which enhances ventilation effectiveness.

**Fig. 6:**
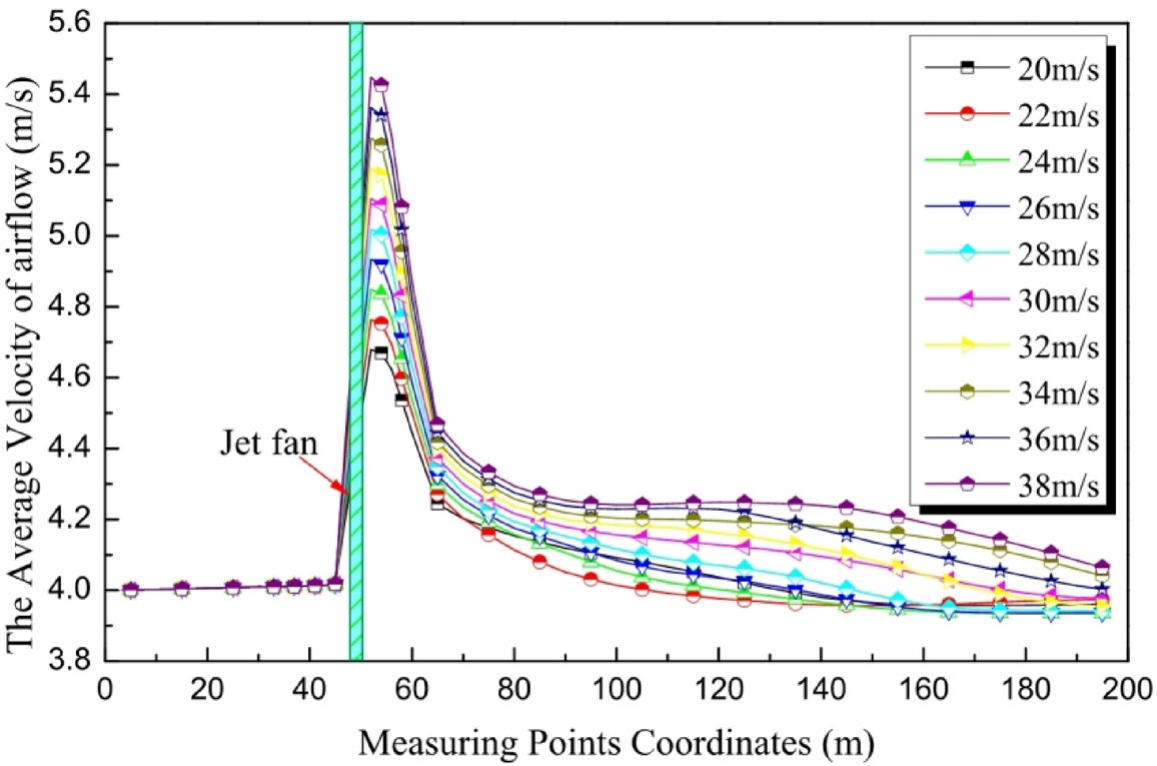
Variation in average velocities at different tunnel cross-sections.

Figure 6 displays the average velocities across the tunnel cross-sections. These profiles follow the same trend as the jet fan centerline data, confirming that the jet magnitude governs the cross-sectional velocity distribution. After pressurization by the jet fan, the average tunnel velocity rises significantly. With increasing downstream distance, it gradually decays, eventually approaching the inlet ventilation speed. The fan imparts thrust to the air at its outlet, injecting a high-kinetic-energy jet into the tunnel and causing a localized velocity increase. As the jet develops and diffuses, its kinetic energy is continuously transferred to the surrounding air. This process gradually homogenizes the velocity profile across the tunnel and ultimately establishes a stable longitudinal ventilation flow.

#### Jet pressure characteristics

**Fig. 7:**
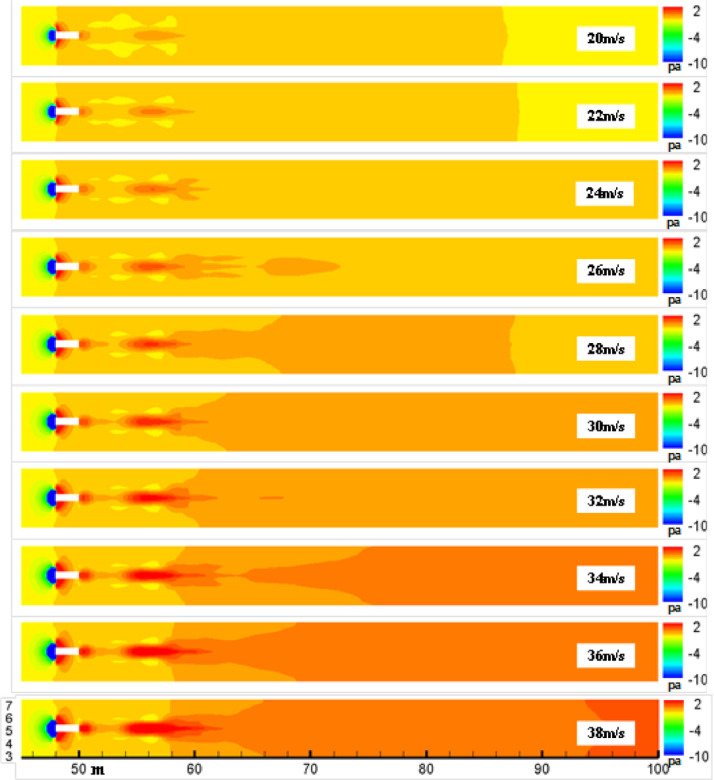
Static pressure distribution under different fan outlet velocities.

The primary mechanism for gas flow in tunnels is the pressure differential, which is markedly influenced by the operation of jet fans. Figure 7 depicts the variation in tunnel static pressure distribution under different jet fan outlet velocities. The results show a distinct negative pressure zone at the fan intake, creating a pressure difference relative to the upstream section that induces longitudinal airflow. Simultaneously, the elevated static pressure at the fan outlet relative to the downstream region generates a pressure gradient that drives the airflow toward the tunnel exit. Functioning as momentum sources, jet fans assist the tunnel air in overcoming resistance, thereby establishing a stable longitudinal flow. As illustrated, a higher fan exit velocity provides greater pressure output, extending both the jet’s coverage and effective range, which results in improved ventilation capacity.

**Fig. 8:**
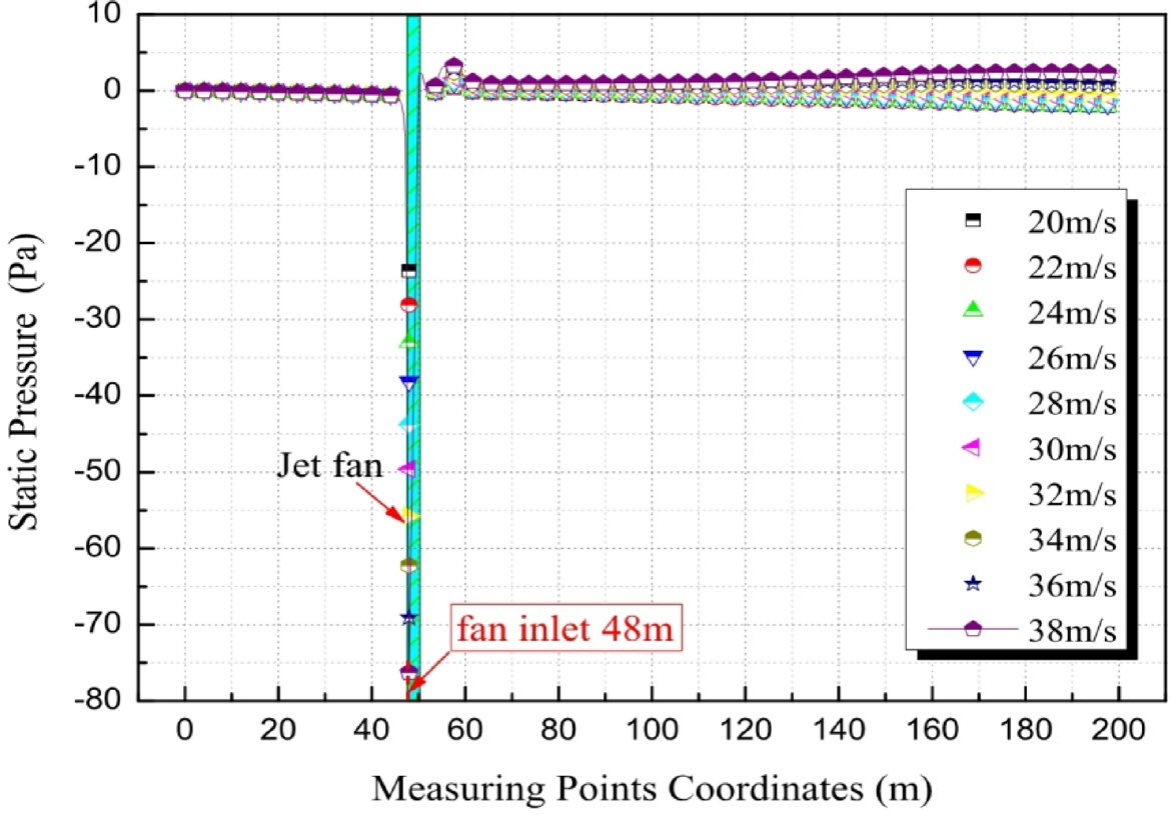
The static pressure of fan axial height along the longitudinal direction.

Figure 8 presents the static pressure distribution along the tunnel axis at various fan outlet velocities. As the velocity increases from 20 m/s to 38 m/s, the static pressure at the fan inlet drops from − 25 Pa to approximately − 80 Pa. Conversely, at fixed downstream locations, the static pressure rises with increasing outlet velocity. The velocity and pressure along the fan axis characterize the jet’s initiation, development, and decay, whereas the cross-sectional averages indicate the overall ventilation capacity and effectiveness of the jet fan.

**Fig. 9:**
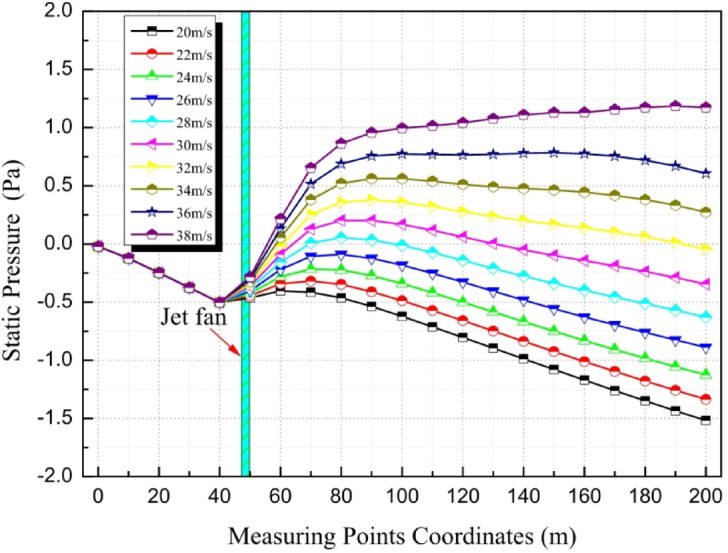
Average static pressure of different sections of tunnel.

Figure 9 shows the variation in cross-sectional average static pressure with outlet velocity. Upstream of the fan, the pressure remains relatively uniform. After being pressurized and accelerated by the jet fan, the airflow in downstream sections shows an overall increase in average static pressure with higher fan velocities. The pressure rise along the tunnel is a key measure of ventilation effectiveness, while the pressure difference across the fan reflects its operational performance. Consequently, increasing the outlet velocity enhances the ventilation efficiency of jet fans in tunnel applications.

#### Jet diffusion characteristics

**Fig. 10:**
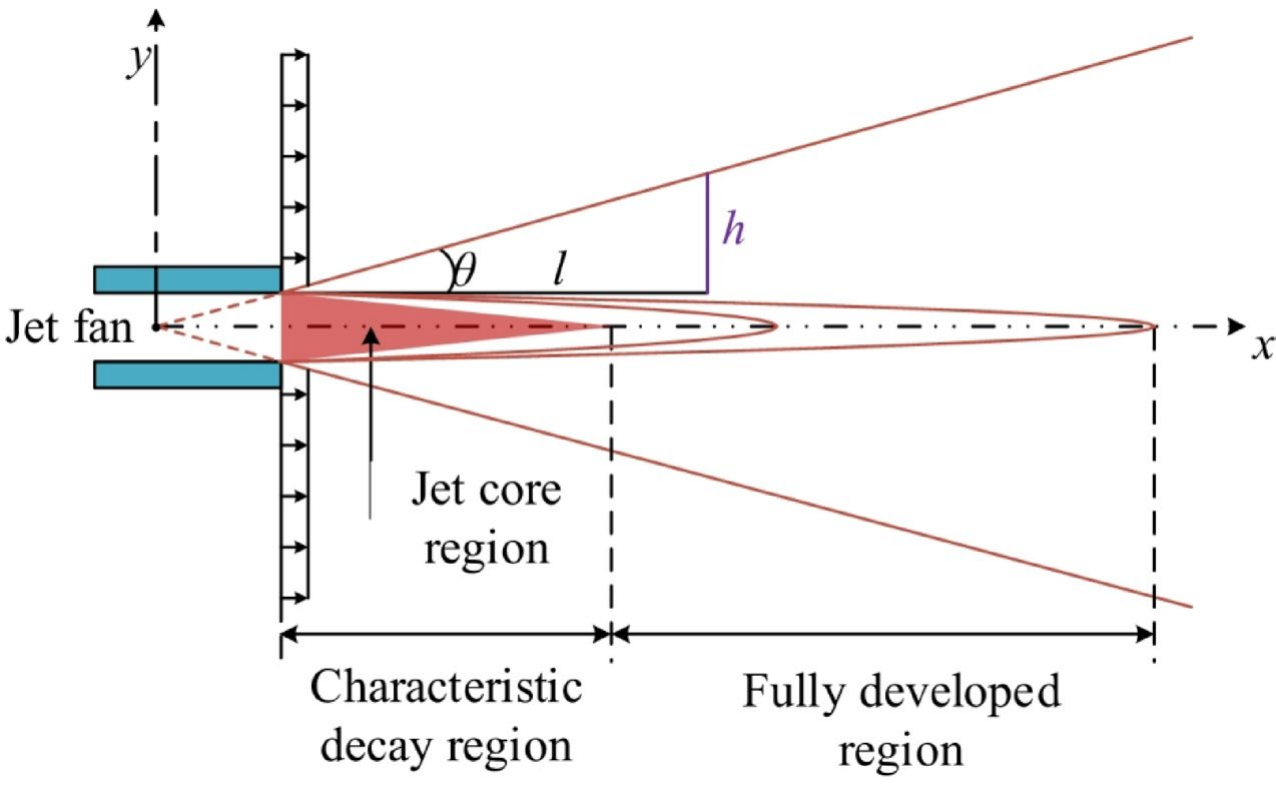
Jet development diagram.

The jet lateral diffusion angle characterizes the transverse spreading rate of the high speed airflow after it is discharged into the tunnel. In this study, this angle is quantified by measuring the lateral spread of the jet boundary at a specific location, as illustrated in Fig. 10. Immediately downstream of the fan outlet, a potential core region forms, within which the jet velocity remains constant and equal to the exit velocity. Beyond this core region, with increasing distance, the jet expands by entraining ambient air, exchanging energy and mass, which results in a progressive decay in velocity. The jet diffusion angle is given by:

6 $$\tan \left( \theta \right)={h \mathord{\left/ {\vphantom {h l}} \right. \kern-0pt} l}$$

**Fig. 11:**
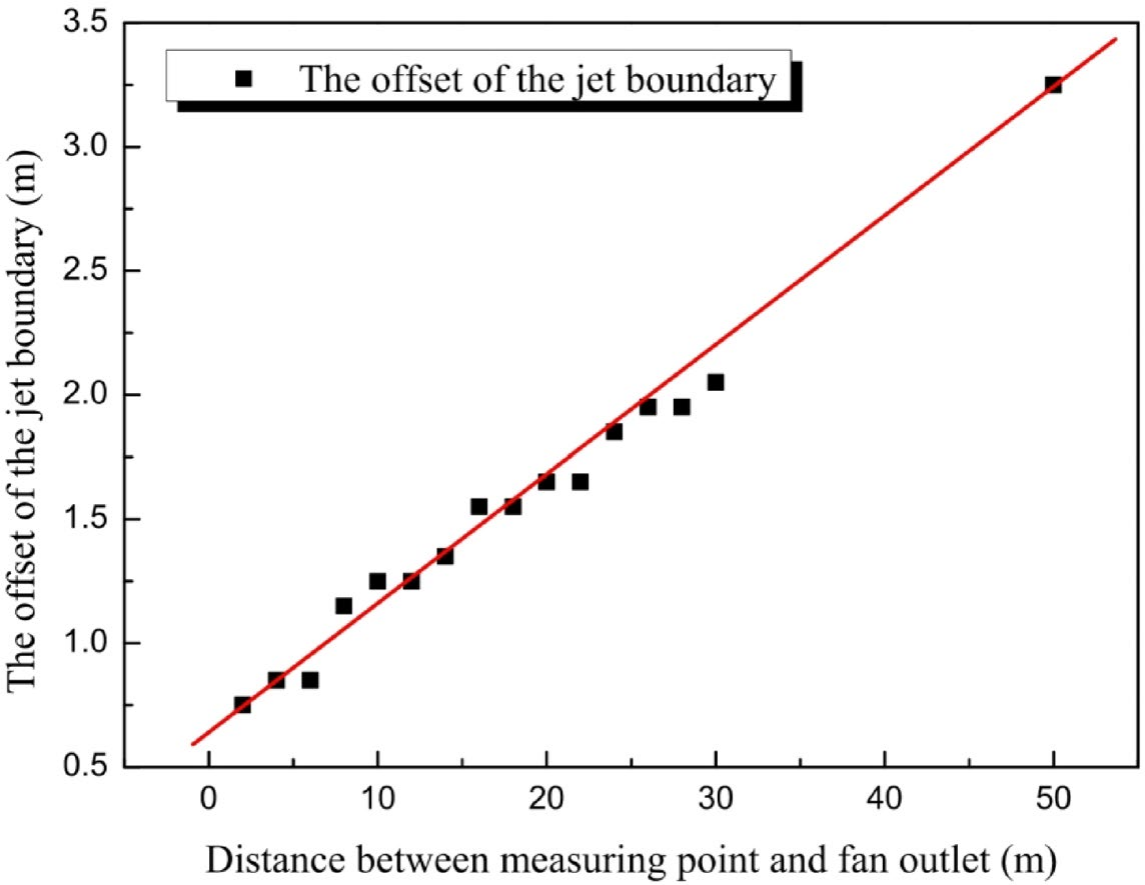
The offset of jet flow.

The jet diffusion angle serves as a key parameter in characterizing axial fan ventilation jets. For improved accuracy, jet angles were computed from measurements taken at multiple downstream locations. Figure 11 presents the lateral boundary displacement for a representative 500 mm fan operating at a 20 m/s outlet velocity. In the absence of confinement or interference, the jet boundary displacement exhibits a linear relationship with the distance from the fan outlet, indicating that the jet diffusion angle can be considered constant under these conditions. Using the same data processing approach, the jet displacement and diffusion angle were determined for different outlet airflow velocities.

**Fig. 12:**
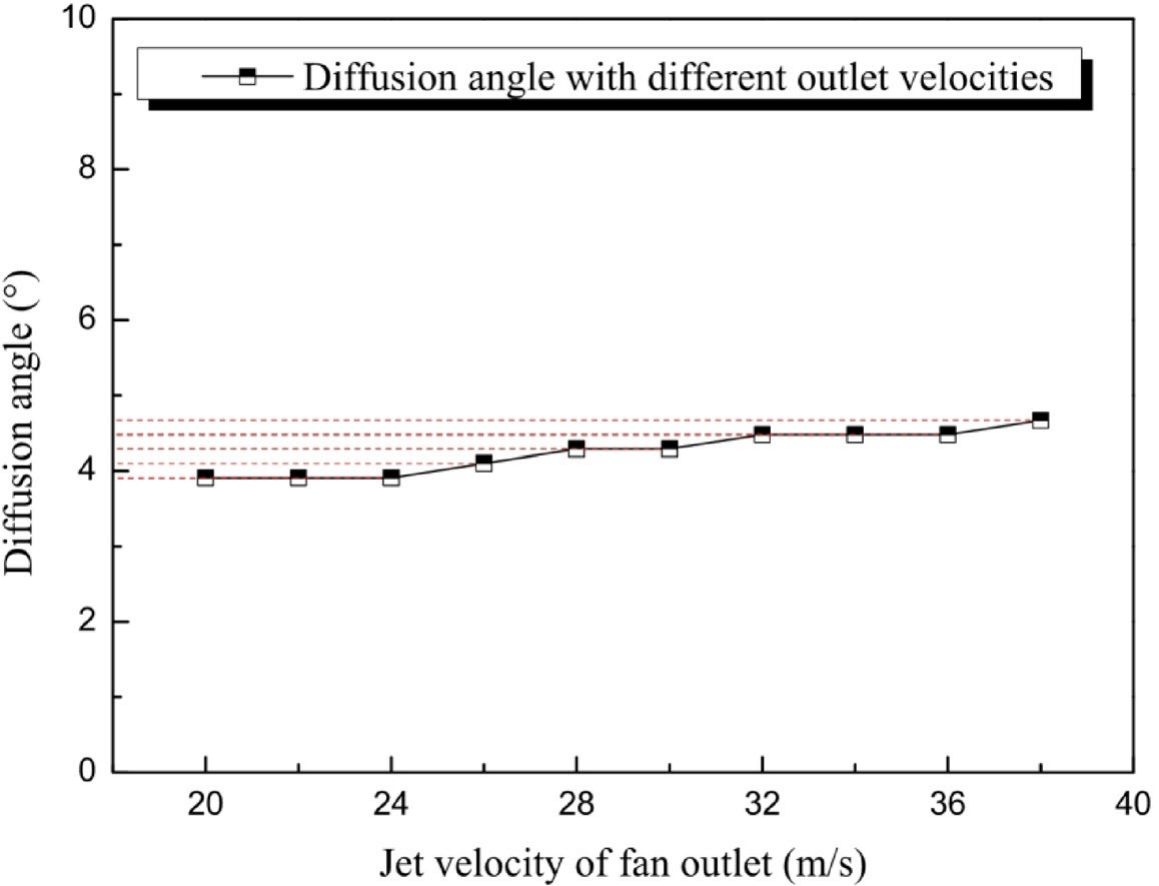
Jet diffusion angle with different fan outlet velocities.

Figure 12 illustrates the variation of the jet diffusion angle with fan outlet velocity. The diffusion angle exhibits a monotonic increase from 3.91° to 4.67° as the outlet velocity rises from 20 m/s to 38 m/s, amounting to a net increase of merely 0.76°. This indicates that while a higher outlet velocity enlarges the diffusion angle, the effect is limited. Therefore, the measured jet diffusion angle serves as a basis for optimizing the placement of fans in tunnels. To minimize ventilation energy consumption and enhance efficiency, the clearance between the fan and the tunnel boundaries should be designed accordingly.

### Effects of fan diameter on tunnel ventilation

Multiple jet fan models and technical parameters are made available for urban tunnel ventilation system design, including variations in exit velocity, axial thrust, impeller diameter, and motor power. At fixed exit velocities, the fan diameter becomes a critical parameter affecting both the ventilation volume and system efficiency. To eliminate interference arising from the ratio of wind tunnel to fan cross-sectional area on airflow velocity and static pressure, the model adopts a standardized axial distance from the fan to the tunnel wall of 10 fan diameters, with the tunnel’s cross-sectional dimensions scaled proportionally to fan diameter.

#### Jet velocity distribution

**Fig. 13:**
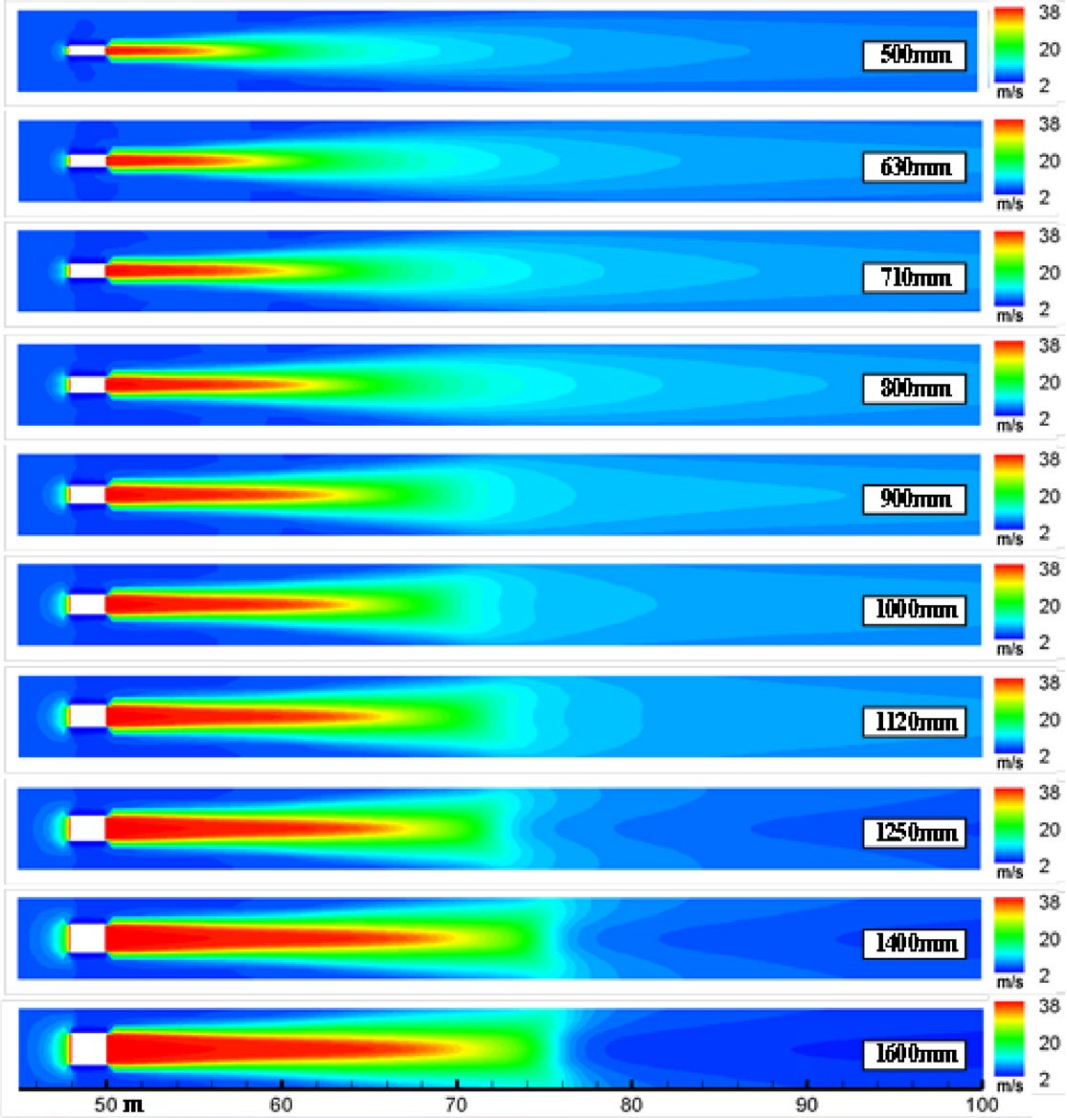
Ventilation jet evolution under varying fan diameters.

Figure 13 shows flow field evolution within the tunnel as fan diameter increases from 500 mm to 1600 mm at a fixed exit velocity of 38 m/s, with the tunnel width maintained at 20 times the fan diameter in all cases. For comparison purposes, each case displays a representative segment of constant width rather than the full tunnel extent.

As illustrated, increasing fan diameter is observed to expand the tunnel jet’s cross-section. During diameter escalation from 500 mm to 800 mm, the jet length continues to extend. However, when the fan diameter exceeds 900 mm, the jet front gradually flattens and even develops a concave shape, which inevitably increases the flow resistance.

From the perspective of airflow velocity distribution, with a constant fan outlet velocity of 38 m/s, the tunnel exhibits effective ventilation when the fan diameter is below 1120 mm, with a well-developed longitudinal airflow ahead of the jet. Once the fan diameter reaches or exceeds 1250 mm, the jet front gradually assumes a “tulip” shape, characterized by lower velocity in the central region and higher velocity along the periphery. This results from increased resistance on larger jets’ advancing fronts, causing axial flow divergence toward peripheral regions and “tulip-shaped” jet formation.

**Fig. 14:**
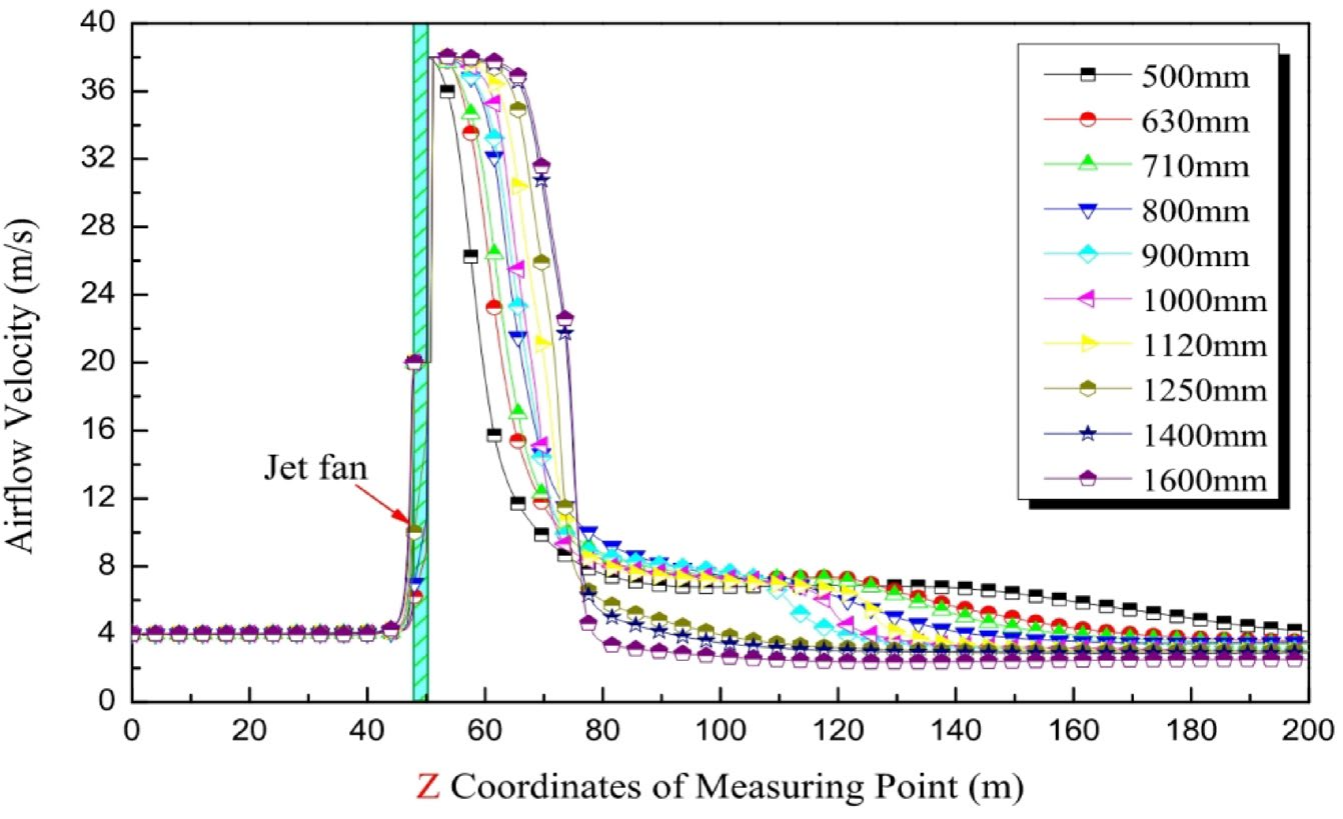
The velocity distribution of the fan axis height under different fan diameters.

**Fig. 15:**
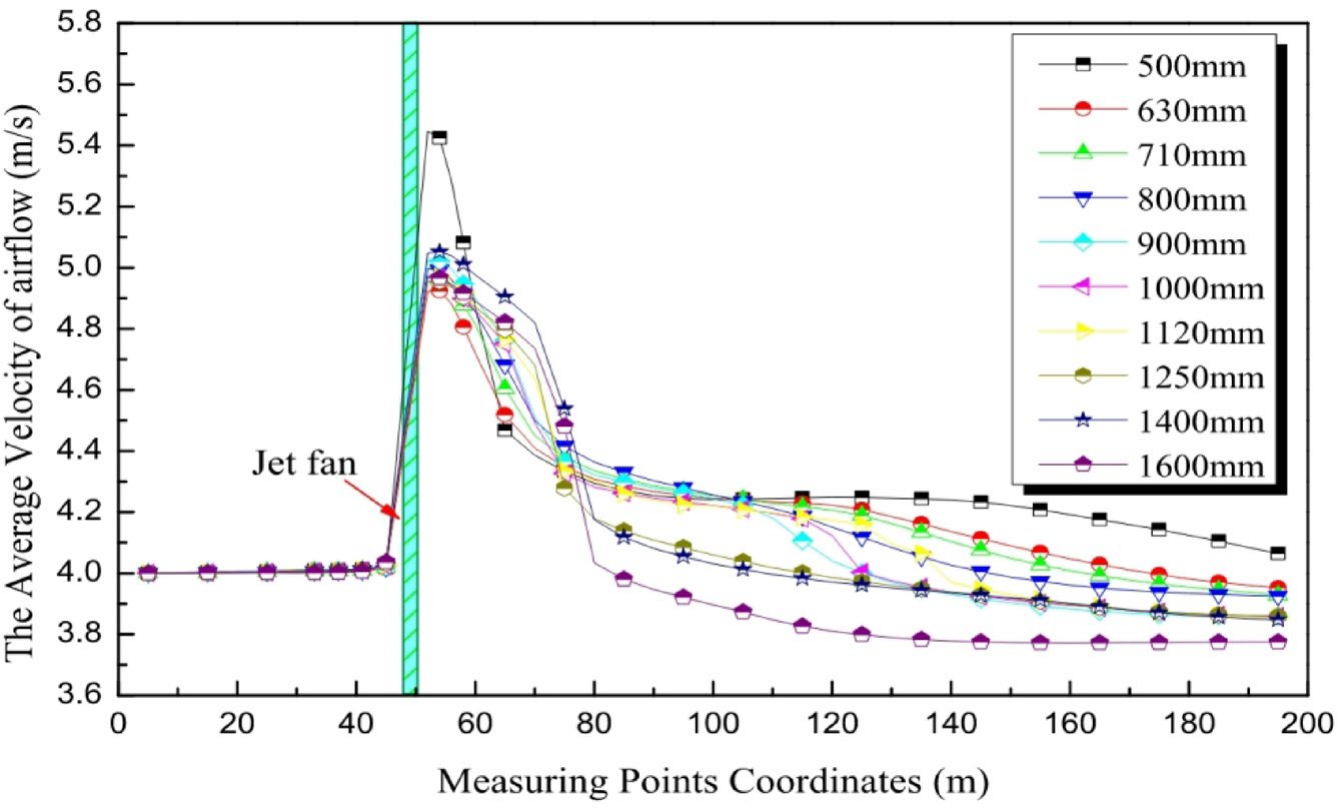
The average velocity of different sections under different fan diameters.

The velocity profiles along the fan centerline and the cross-sectional average velocities at different longitudinal positions are illustrated in Figs. 14 and 15, respectively, for varying fan diameters. For smaller fan diameters, the airflow velocity decays rapidly downstream of the fan exit. In contrast, configurations featuring larger fan diameters sustain a longer high-velocity jet region, indicating that larger fans enhance both pressurization and flow acceleration within the tunnel. A key finding is that for fan diameters of 1250 mm or greater, a sharp velocity decay occurs along the axis at about 75–80 m downstream. This phenomenon becomes more evident with larger diameters, indicating substantial jet divergence and consistent with the tulip-shaped jet front observed in Fig. 13.

The average cross-sectional velocity in the tunnel reflects the ventilation effectiveness of the jet fan to some extent. As shown in Fig. 15, when the fan diameter exceeds 1250 mm, the average cross-sectional velocity decreases significantly beyond 80 m downstream. Beyond 120 m, the average velocity exhibits a decreasing trend with increasing fan diameter. This behavior indicates that during the propagation and development of the high speed jetflow in the tunnel, energy is continuously exchanged with the surrounding air through mechanisms such as dragging, entrainment, and pushing. These processes promote a more uniform and stable velocity distribution across the tunnel section, but also result in substantial energy loss. Larger fan diameters are more prone to jet front divergence. The reduction in average cross-sectional velocity beyond 80 m with increasing fan diameter further confirms that deformation and divergence of the jet front lead to significant ventilation energy loss.

#### Static pressure distribution in tunnel

**Fig. 16:**
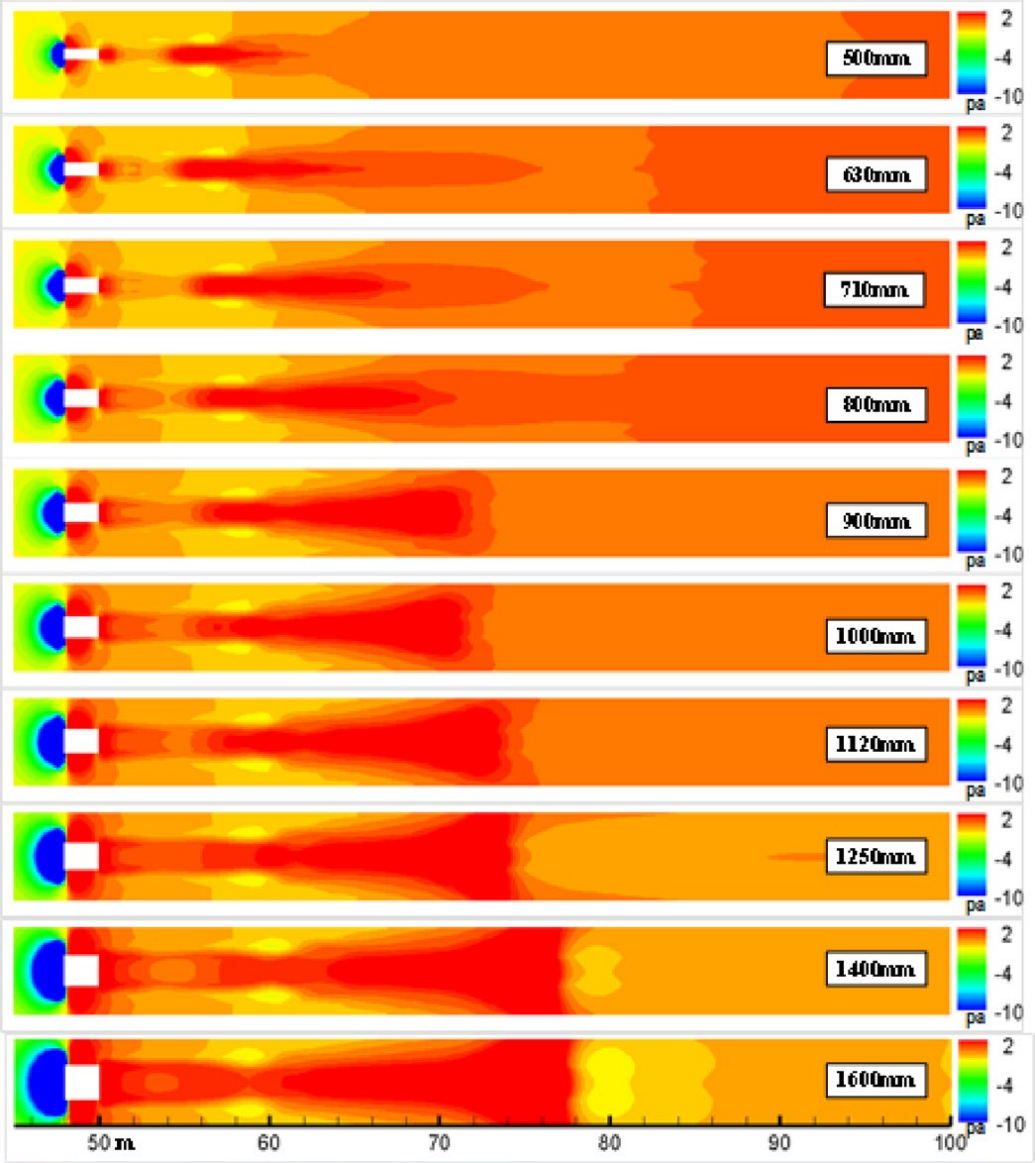
The static pressure distribution in the tunnel under different fan diameters.

The influence of fan diameter variation on the static pressure distribution in the tunnel differs from that of outlet velocity changes. Figure 16 illustrates the static pressure variation under different fan diameters. It can be observed that the low-pressure zone formed at the fan inlet expands with increasing fan diameter, and the downstream pressure rise grows in magnitude and spatial extent. Similar to the velocity field, the front region of higher static pressure gradually flattens and even becomes concave with larger fan diameter. This analogous behavior further demonstrates the coupling relationship between the static pressure field and the flow field.

**Fig. 17:**
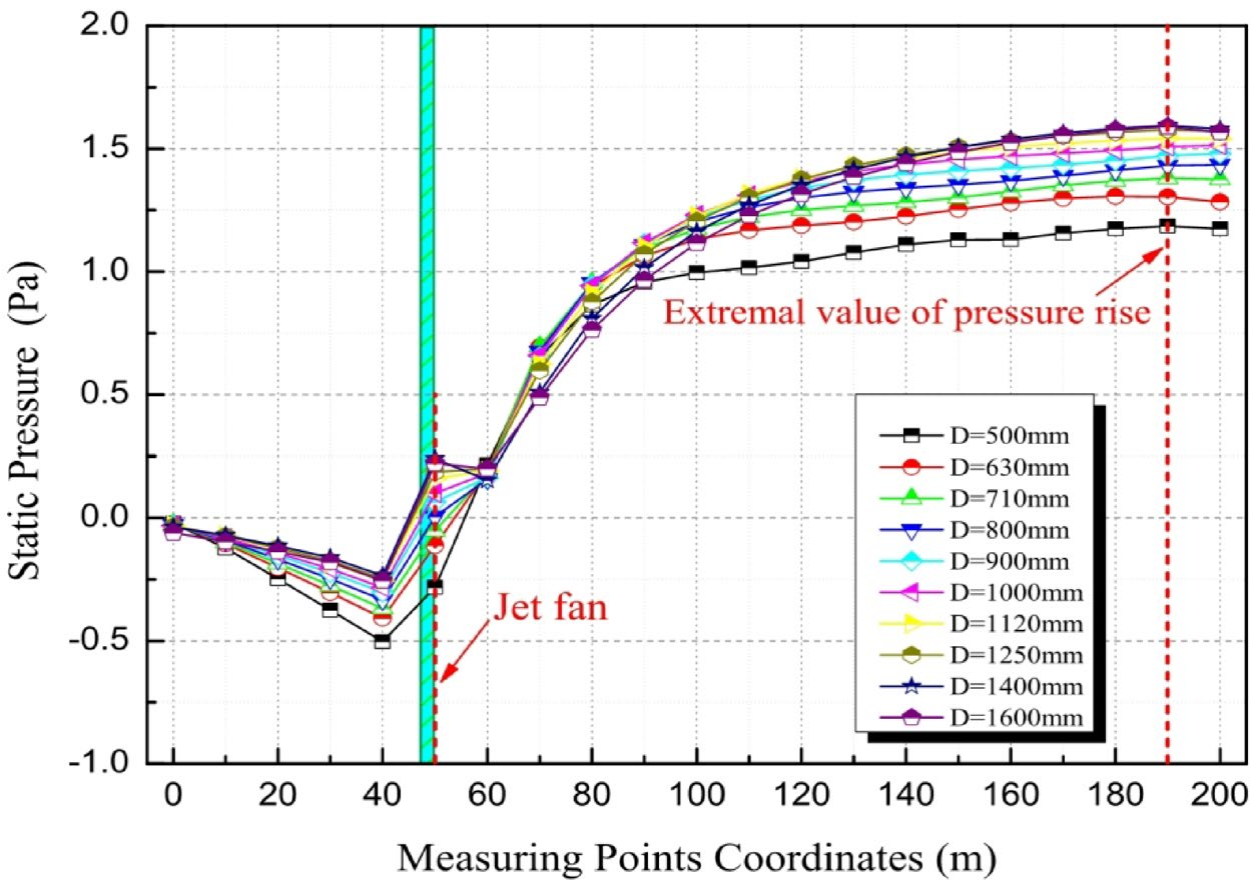
The average static pressure of sections under different fan diameters.

Figure 17 illustrates the variation in average static pressure across different tunnel cross-sections as a function of jet fan diameter. Minimal sensitivity to fan diameter is observed within the 0–80 m zone from the fan inlet, whereas positions closer to the tunnel exits exhibit progressively lower static pressures with increasing fan diameter. This suggests that smaller-diameter fans promote more favorable longitudinal airflow development and greater resistance-overcoming capability. In contrast, larger-diameter fans enhance overall ventilation capacity and local airflow velocity near the tunnel exits, however, they also induce stronger resistance-driven flow divergence and higher energy dissipation during jet propagation.

**Fig. 18:**
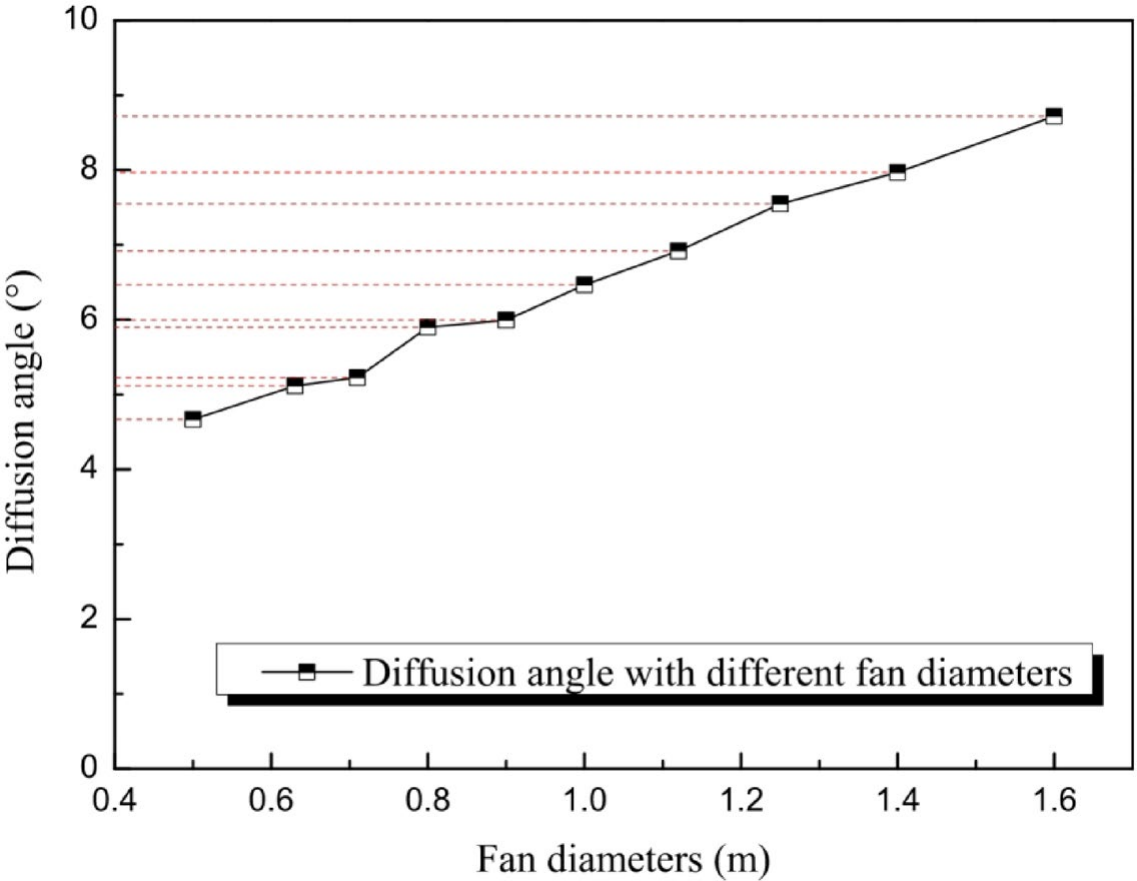
Jet diffusion angle under different fan diameters.

#### Jet diffusion characteristics

Jet diffusion angles directly indicate airflow development generated by the fan within the tunnel. Figure 18 illustrates the relationship between the jet diffusion angle and the fan diameter. As the fan diameter increases from 500 mm to 1600 mm, the jet diffusion angle increases approximately linearly from 4.67° to 8.7°. Larger diameters produce greater diffusion angles. To avoid significant ventilation energy loss caused by high speed jets impinging on the tunnel roof and sidewalls, it is essential to maintain an adequate distance between the fan and the tunnel walls during installation.

### Pressure rise coefficient

The pressure rise within a tunnel is a key indicator of the ventilation capacity of jet fans. To evaluate the ventilation performance of jet fans with different diameters and outlet velocities, the pressure increase generated during operation is used as a quantitative measure. When a jet fan is operating, the negative pressure zone formed in front of the fan induces upstream airflow to move downstream, while the elevated pressure behind the fan propels the air forward. The pressure rise capability of the jet fan can be quantified as the difference between the average static pressure at a downstream cross-section and the minimum static pressure measured immediately upstream. The corresponding expression^22,28^ is defined as:

7 $$\Delta {P_j}={p_t} - {p_{\hbox{min} }}$$

The tunnel ventilation efficiency can be quantified by the fan pressure rise coefficient $\eta$^10^, defined as the ratio of actual pressure rise generated by jet fans to the theoretical pressure rise that would be delivered under ideal conditions. This coefficient is expressed as:

8 $$\eta =\frac{{\Delta {p_{j-a}}}}{{\Delta {p_{j - t}}}}$$

9 $$\Delta {p_{j-a}}={p_{\hbox{max} }} - {p_{\hbox{min} }}$$

10 $$\Delta {p_{j - t}}=n\rho v_{j}^{2}\frac{{{A_j}}}{{{A_t}}}\left( {1 - \frac{{{v_t}}}{{{v_j}}}} \right)$$

**Fig. 19:**
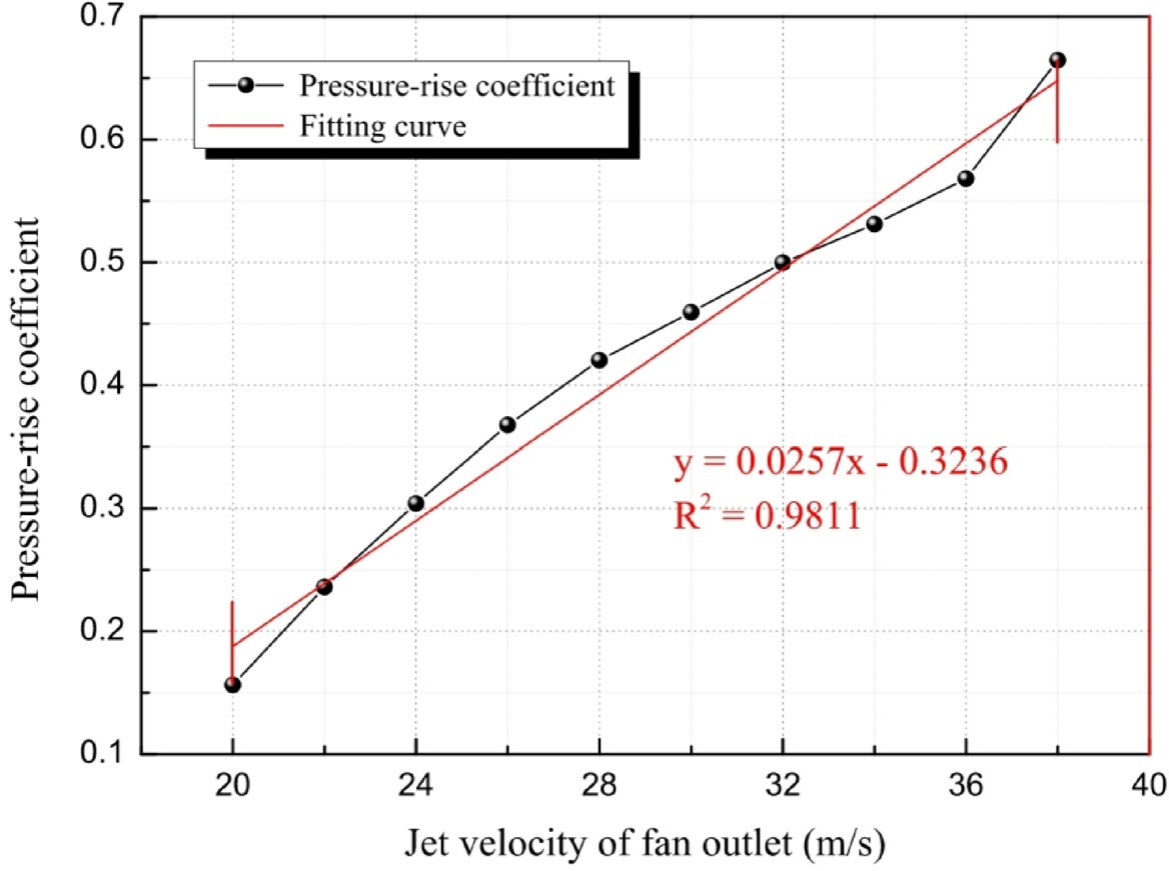
Pressure rise coefficient versus jet fan outlet velocity.

**Fig. 20:**
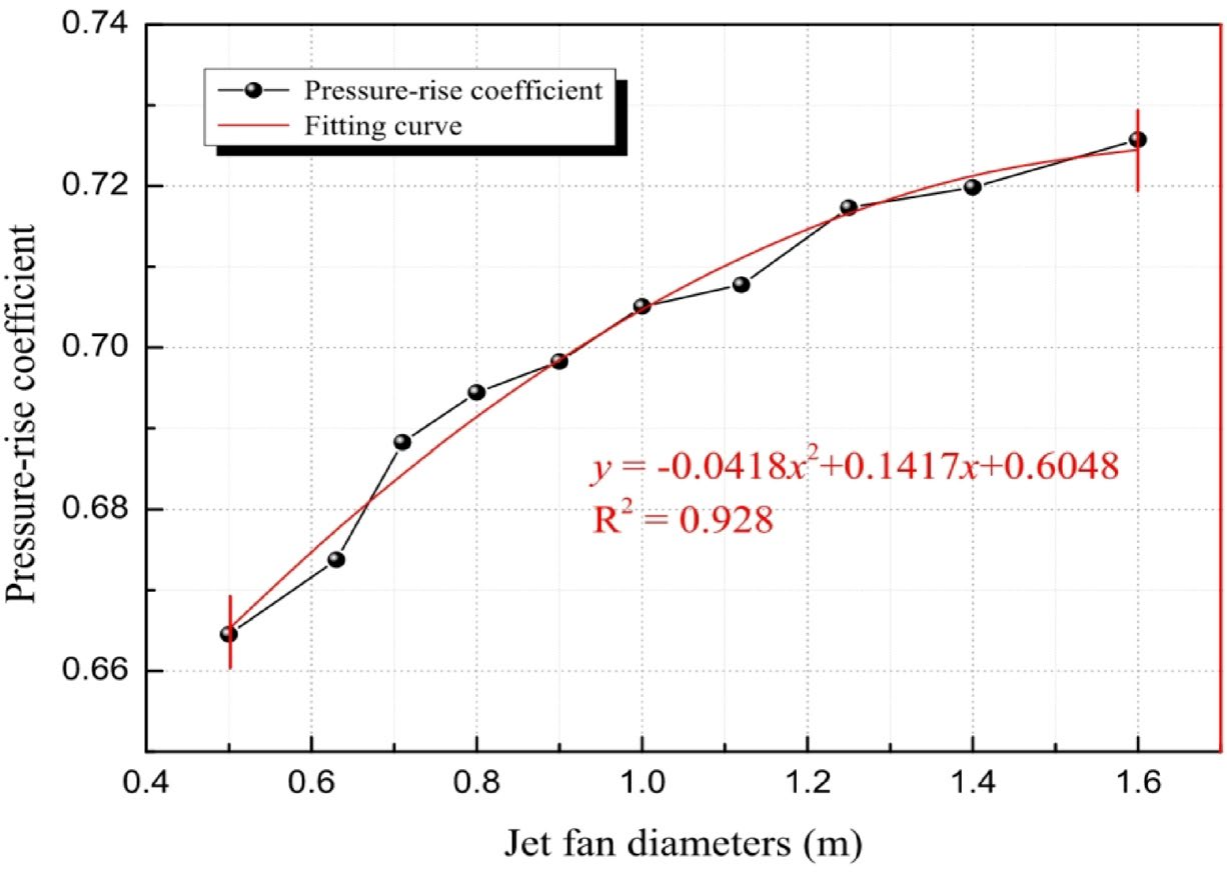
Pressure rise coefficient versus jet fan diameter.

Based on formulas (7) to (10), the numerical simulation data of outlet velocity and diameter are derived and calculated to obtain Figs. 19 and 20. Figure 19 shows the variation of the pressure rise coefficient when the fan diameter is 500 mm and the outlet velocity increases from 20 m/s to 38 m/s. It can be observed that as the outlet velocity increases, the pressure rise coefficient increases significantly from 16% to 66%, which indicates that increasing the outlet velocity of the jet fan can effectively improve the ventilation efficiency of the large-section tunnel. Under the same outlet velocity (38 m/s), as the fan diameter increases from 500 mm to 1600 mm, the pressure rise coefficient increases from 66% to 73%, which has a significant quadratic positive correlation with the fan diameter (see Fig. 20). Therefore, increasing the fan diameter can improve ventilation efficiency to a certain extent; however, the pressure rise coefficient will reach an extreme value as the fan diameter increases further.

## Conclusion

In this paper, the influence mechanisms of jet fan outlet velocity and diameter on ventilation efficiency were investigated and analyzed using Computational Fluid Dynamics (CFD). A series of tunnel ventilation models were established with a fixed ratio of fan diameter (D) to tunnel width (L) of 1:20. A total of 19 simulation cases were designed by varying the jet outlet velocity (ranging from 20 m/s to 38 m/s) and the fan diameter (ranging from 500 mm to 1600 mm) as independent variables. Based on the analysis of the simulated jet characteristics, main conclusions are as follows:

(1) Increasing the outlet velocity of jet fans could significantly promote the propagation of airflow inside tunnels, while enhancing the entrainment effect and propulsion effect of jet flow. When the outlet velocity was increased from 20 m/s to 38 m/s, the jet diffusion angle changed slightly, and the pressure rise coefficient increased from 16% to 66%. A good linear positive correlation was observed between the pressure rise coefficient and the fan outlet velocity. Therefore, increasing the outlet velocity is one of the effective approaches to enhance the ventilation capacity in tunnel.

(2) Increasing the diameter of the fan could significantly expand the jet diffusion angle. This not only facilitated the uniform distribution of airflow, but also effectively increased the ventilation rate, thereby improving the overall ventilation performance. At the outlet velocity of 38 m/s, when the fan diameter was increased from 500 mm to 1600 mm, the jet length and diffusion angle increased synchronously, the pressurization and acceleration effects of the jet fan were significantly enhanced, and the fan pressure rise coefficient increased from 66% to 73%. The pressure rise coefficient exhibited a quadratic correlation with the fan diameter. Increasing the diameter of jet fan could improve the ventilation efficiency within a certain range; however, with the continuous increase of the fan diameter, the magnitude of improvement in ventilation efficiency would gradually attenuate.

(3) For the tunnel equipped with longitudinal jet ventilation system, under the premise of ensuring the safety of personnel and equipment, increasing the outlet velocity and fan diameter could effectively improve the ventilation efficiency, thereby reducing ventilation energy consumption. Tunnel structural parameters are important factors affecting ventilation coefficient. For different tunnel projects, CFD can be used to optimize fan selection. The research results of this paper can provide a theoretical reference for the optimal design of tunnel ventilation systems.

## Data Availability

The authors confirm that the data supporting the findings of this study are available within the article.

## List of symbols

${A_j}$: Jet fan cross-sectional area (m^2^)
${A_t}$: Tunnel cross-sectional area (m^2^)
$${b_0}$$: nozzle radius (m)
$${C_{{\varepsilon _1}}},{C_{{\varepsilon _2}}}$$: Model constants
*D*: Fan diameter (mm)
${g_i}$: Mass force (N/kg)
${G_k}$: Energy productionkdue to the turbulent stress (Pa/s)
*h*: Full diffusion width of the compound jet (m)
*H*: Height of the tunnel (m)
*k*: Turbulent kinetic energy (J)
*l*: Distance from the nozzle to the downstream cross-section (m)
*L*: Width of the tunnel (m)
*n*: Number of jet fan units
$\Delta {P_j}$: Pressure rise due to jet fans (Pa)
${P_{\hbox{min} }}$: Minimum value of the average static pressure among all cross-sections (Pa)
${p_{\hbox{max} }}$: Maximum value of average static pressure among all cross-sections in tunnel (Pa)
${P_t}$: Average static pressure at the test cross-section (Pa)
$\Delta {P_{j - a}}$: Actual pressure rise caused by jet fan (Pa)
$\Delta {P_{j - t}}$: Theoretical pressure rise magnitude of jet fans (Pa)
*t*: Time variable (s)
${u_0}$: Outlet velocity of the turbulent jet (m/s)
${u_m}$: Speed on the centerline of the jet (m/s)
$${u^{\prime}_i},{u^{\prime}_j}$$: Fluctuating velocities (m/s)
${\bar {u}_i},{\bar {u}_j}$: Time-average velocities (m/s)
${v_j}$: Jet fan outlet velocity (m/s)
${v_t}$: Designed ventilation wind speed for the tunnel (m/s)
$$x,y$$: Cartesian coordinates (m)

## Greek symbols

$\varepsilon$: Turbulent dissipation rate (J/s)
$\eta$: Pressure rise coefficient
$$\mu$$: Dynamic viscosity [N/(m^2^·s)
$${\mu _t}$$: Turbulent viscosity [N/(m^2^·s)
$\theta$: Jet diffusion angle (°)
$\rho$: Air density (kg/m^3^)
$${\sigma _k},{\sigma _\varepsilon }$$: Model constants
$\omega$: Specific dissipation rate (s^-1^)
